# Genetic Diversity of Near Genome-Wide Hepatitis C Virus Sequences during Chronic Infection: Evidence for Protein Structural Conservation Over Time

**DOI:** 10.1371/journal.pone.0019562

**Published:** 2011-05-05

**Authors:** Hui Li, Austin L. Hughes, Nazneen Bano, Susan McArdle, Stephen Livingston, Heike Deubner, Brian J. McMahon, Lisa Townshend-Bulson, Rachel McMahan, Hugo R. Rosen, David R. Gretch

**Affiliations:** 1 Department of Laboratory Medicine, University of Washington Medical Center, Seattle, Washington, United States of America; 2 Department of Biological Sciences, University of South Carolina, Columbia, South Carolina, United States of America; 3 Liver Disease and Hepatitis Program, Alaska Native Tribal Health Consortium, Anchorage, Alaska, United States of America; 4 Department of Pathology, University of Washington Medical Center, Seattle, Washington, United States of America; 5 Division of Gastroenterology and Hepatology, Department of Medicine, University of Colorado School of Medicine, Aurora, Colorado, United States of America; 6 Department of Medicine, University of Washington Medical Center, Seattle, Washington, United States of America; University of Montreal, Canada

## Abstract

Infection with hepatitis C virus (HCV) is one of the leading causes of chronic hepatitis, liver cirrhosis and end-stage liver disease worldwide. The genetics of HCV infection in humans and the disease course of chronic hepatitis C are both remarkably variable. Although the response to interferon treatment is largely dependent on HCV genotypes, whether or not a relationship exists between HCV genome variability and clinical course of hepatitis C disease still remains unknown. To more thoroughly understand HCV genome evolution over time in association with disease course, near genome-wide HCV genomes present in 9 chronically infected participants over 83 total study years were sequenced. Overall, within HCV genomes, the number of synonymous substitutions per synonymous site (*d_S_*) significantly exceeded the number of non-synonymous substitutions per site (*d_N_*). Although both *d_S_* and *d_N_* significantly increased with duration of chronic infection, there was a highly significant decrease in *d_N_*/*d_S_* ratio in HCV genomes over time. These results indicate that purifying selection acted to conserve viral protein structure despite persistence of high level of nucleotide mutagenesis inherent to HCV replication. Based on liver biopsy fibrosis scores, HCV genomes from participants with advanced fibrosis had significantly greater *d_S_* values and lower *d_N_*/*d_S_* ratios compared to participants with mild liver disease. Over time, viral genomes from participants with mild disease had significantly greater annual changes in *d_N_*, along with higher *d_N_*/*d_S_* ratios, compared to participants with advanced fibrosis. Yearly amino acid variations in the HCV p7, NS2, NS3 and NS5B genes were all significantly lower in participants with severe versus mild disease, suggesting possible pathogenic importance of protein structural conservation for these viral gene products.

## Introduction

Hepatitis C virus (HCV) is a single-strand, positive-sense RNA virus classified within the *Hepacivirus* genus of the *Flaviviridae* family. Approximately 3 million people in the United States and 170 million people worldwide are infected with HCV [1]. Upon HCV infection, up to 80% individuals will develop persistent viraemia and chronic hepatitis, which potentially leads to liver cirrhosis, hepatocellular carcinoma, end-stage liver disease, and liver failure [2]. Cofactors such as alcohol intake, obesity, HIV coinfection and underlying liver-related diseases also accelerate the progression of hepatitis C to cirrhosis [3]. The standard treatment for patients with chronic hepatitis C is a combination of ribavirin and pegylated interferon alpha, which is effective in eradication of HCV in approximately 50% of patients despite significant side effects [4].

The ∼9.6 kb HCV genome encodes 5′ and 3′ untranslational regions, plus a single open reading frame that is subsequently processed into three structural proteins (Core, E1, and E2) and seven non-structural proteins (p7, NS2, NS3, NS4A, NS4B, NS5A and NS5B). As occurs with many other RNA viruses, HCV exhibits a considerable degree of sequence variation over the whole genome. Six major genotypes have been described that share 70–80% nucleotide identity with one another, along with more than 80 subtypes that share 80–90% nucleotide identities within these genotypes [5]. In infected individuals, HCV circulates as a population of closely related yet distinguishable variants with less than 10% differences at the nucleotide level [6]. The distribution of the variant population dynamically deviates through adaptive or neutral evolution [7]. Several regions on the HCV genome have been extensively studied in association with therapeutic resistance and/or clinical outcome, including the hypervariable region 1 (HVR1) of E2 [8], [9], [10], the alpha interferon sensitivity determining region of NS5A [11], [12], and the RNA polymerase of NS5B [13]. Although genetic variation of individual viral protein domains may be highly significant between patient groups, multi-domain and whole genome analyses are needed to facilitate understanding of the role viral diversification plays with respect to underlying disease mechanisms [14].

The present report describes genetic analysis of near genome-wide HCV genomes isolated from 9 chronically infected participants recruited into the well-characterized Alaska Native cohort [15]. All 9 participants were infected with HCV genotype 1, and had liver biopsies to document progression or stability of hepatic fibrosis. The median follow up interval post-primary infection was 23 years, and the interval between specimens ranged from 5–21 years. HCV whole genome evolution was compared according to estimated years of infection and disease progression, making this the first population-based, longitudinal study of genome-wide HCV genome evolution during chronic infection and disease.

## Materials and Methods

### Ethics Statement

The study group was comprised of 9 participants from the Alaska Native cohort as described previously [15]. All participants gave written informed consent in accordance with the Institutional Review Board (IRB) requirements by the Alaska Area IRB, the Centers for Disease Control and Prevention, and the University of Washington. All three IRBs specifically approved this study (Text S1).

### Patients and serum specimens

All participants were chronically infected with HCV genotype 1, negative of HIV and HBV coinfections, and naïve to interferon or ribavirin treatment. The participants were all therapy naïve because they either did not meet the criteria for treatment outlined in the AASLD Practice Guidelines (mile disease), or they had a treatment contraindication (e.g., severe depression), or they chose not to be treated due to drug toxicity concerns. Patient interview information was used to estimate the years of HCV infection and the risk exposure categories (intravenous drug use, before-1992 blood transfusion, or others). Phlebotomy was scheduled every 3–6 months to monitor the hepatic aminotransferase activities [16], [17], and serum specimens were collected at selected time points and stored at −30°C for future analysis. HCV viral RNA levels were determined by branched DNA assay (Bayer Corporation, Tarrytown, NY) and/or quantitative PCR (Roche Diagnostic Systems, Branchburg, NY) at the University of Washington. HCV genotype was determined by restriction fragment length polymorphism analysis of the 5′-UTR region and confirmed by probe hybridization of the Core/E1 region [18], [19]. Disease progression was inferred from liver biopsies (in average 2 biopsies per patient) that were evaluated by the study pathologist (HD) at the University of Washington, who was blinded to demographic and clinical data, according to the Ishak and Knodell scoring system [20], [21].

### Viral RNA extraction and sequencing

**Figure 1:**
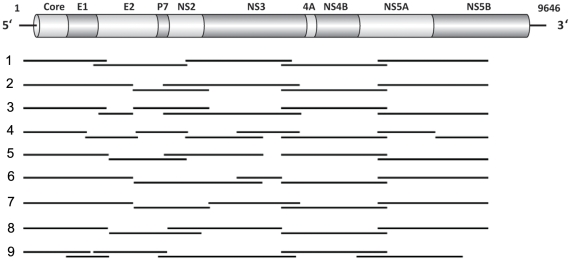
Strategies used to amplify the near genome-wide HCV genome from 9 participants. The top bar illustrates the HCV genomic organization with 10 encoded proteins (Core to NS5B) flanked by the 5′ and 3′ UTRs. Lines below represent the overlapping fragments amplified by reverse transcription PCR for each participant (PCR primers listed in Table S1). The nucleotide numbering is based on the sequence of HCV H77 strain (Genbank assession no. NC_004102).

Viral RNA was extracted from 140 µL serum using the QIAamp viral RNA Mini Kit (QIAGEN Inc., Valencia, CA) and reverse transcribed with the Superscript III First-Strand Synthesis System and random hexamers (Invitrogen Corp., Carlsbad, CA). Amplification of the near genome-wide HCV genome was performed with 5–8 PCRs using the Platinum *Taq* DNA Polymerase High Fidelity (Invitrogen Corp., Carlsbad, CA) and multiple sets of primers (Table S1) as illustrated in Figure 1. The PCR recycling conditions were as follows: 94°C for 2 min, followed by 35 cycles of 94°C for 30 s, 55°C for 30s, 68°C for 1 min/kb, and a final extension of 68°C for 10 min. The PCR products were further cloned using the TOPO TA cloning kit (Invitrogen Corp., Carlsbad, CA), and three colonies were picked for bidirectional sequencing using the PCR primers and additional internal primers (Table S2). The sequences were aligned using the MacVector software (version 9.5, MacVector Inc., Cary, NC) for each amplified fragment, with the primer sequences removed. Sequences generated in this study have been deposited in GenBank under accession numbers HQ113464 through HQ113761. Phylogenetic trees based on the nucleotide and derived amino acid sequences of the Core region were constructed and included in Figure S1.

### Diversity and entropy analysis

The number of synonymous substitutions per synonymous site (*d_S_*) and the number of non-synonymous substitutions per non-synonymous (*d_N_*) site were estimated using the MEGA and SNAP program (http://www.hiv.lanl.gov) [22] based on the Nei and Gojobori's method [23]. In preliminary analyses, the methods of Li [24] and Yang and Nielsen [25] yielded essentially identical results, as expected because the number of substitutions per site was low in this case [26]. The resulting values were used to compute the mean of all pairwise synonymous substitution values (synonymous nucleotide diversity; *π_S_*) and the mean of all pairwise non-synonymous substitution values (non-synonymous nucleotide diversity; *π_N_*). The Shannon entropy per site was determined from the deduced amino acid sequences of each amplified fragment and each viral gene using the Entropy program on the Los Alamos National Laboratories website (http://hcv.lanl.gov/content/sequence/ENTROPY/entropy_one.html) [27].

### Statistical tests

Since each of these amplified fragments was sampled separately from each patient at each time point, they were treated as independent samples for purposes of statistical analyses. The Kruskal-Wallis and Mann-Whitney tests were used to compare median values of *π_S_*, *π_N_*, *π_N_*/*π_S_*, and the Shannon entropy across categories of patients. The Spearman's correlation coefficient (r) was calculated to determine the relationship between measures of sequence diversity and the estimated year of infection. P-values of less than 0.05 were considered to be statistically significant. The statistical tests were performed using the PASW Statistics program 18.0 (SPSS Inc., Chicago, IL).

## Results

### Sample characteristics

The characteristics of the nine patients included in the study are summarized in Table 1. Two serum specimens from each participant were analyzed by near genome-wide sequencing, with an interval between the early and late collections ranging from 5–21 years (median = 7 years, sum = 83 years). The early collections were at 2–32 years after the initial year of infection (median = 13 years), while the late collections were at 13–38 years after the initial year of infection (median = 23 years). All participants were infected with HCV genotype 1 (8 genotype 1a and 1 genotype 1b), with mixed-infection or super-infection ruled out by probe hybridization of the Core/E1 region [19]. HCV RNA level was estimated as the range of all the documented RNA levels for each participant (n = 3–9) over 2–3 decades, from the initial date of patient enrollment to the year of the late serum collection. The participant 1 showed the greatest variation of RNA levels (log_10_ IU/ml = 4.7–7.3) among the included participants during chronic infection. Eight of the nine participants underwent at least one liver biopsy on, or in the case of some participants with mild disease, after the year of serum collection. Knodell fibrosis stage 0–1 was considered as the indicator of mild liver disease, and Knodell fibrosis stages 3–4 were considered as severe liver disease. The hepatic aminotransferase levels (aspartate aminotransferase, AST; alanine aminotransferase, ALT; alpha-fetoprotein, AFP) were estimated as the means of the documented serial aminotransferase levels (n = 1–8) for each participant at the year of serum collection. The AST/ALT ratio was slightly above 1 at the early time point for the participant 4 (fibrosis stage 0) and at the late time point for the participants 3 (fibrosis stage 3), 4 (fibrosis stage 0–1) and 8 (fibrosis stage 1). The highest AST/ALT ratio was observed at the early time point for the participant 8 (fibrosis stage 1; AST/ALT = 2.1) with ALT below the normal level (ALT = 32 U/L). The AFP level was above 8 at the early time point for the participants 1 (fibrosis stage 4) and 3 (fibrosis stage 1) and at the late time point for the participant 3 (fibrosis stage 3).

**Table 1:** Clinical information of the study participants.

Participant	Gender	Age at infection	Risk exposure^a^	HCV genotype	HCV RNA range (log_10_ IU/ml)^b^	Early specimen					Late specimen				
						Years infected	KnodellFibrosis stage	AST (U/L)^c^	ALT (U/L)^c^	AFP (ng/ml)^c^	Years infected	KnodellFibrosis stage	AST (U/L)^c^	ALT (U/L)^c^	AFP (ng/ml)^c^
1	M	16	IDU	1a	4.7–7.3	26	4	176	258	8.9	38	4	47	72	5.2
2	F	44	IDU	1a	5.8–6.7	3	1	50	62	1.5	14	1	38	43	3.7
3	F	30	Others	1a	4.7–6.2	15	1	57	71	9.0	25	3	32	30	14.0
4	F	29	Others	1a	5.7–6.3	7	0	52	49	2.0	13	0–1	43	38	3.2
5	M	25	IDU	1a	6.0–7.3	29	N/A	25	32	2.9	35	N/A	24	33	3.7
6	M	25	IDU	1a	6.1–7.0	13	1	55	126	3.8	20	N/A	30	67	5.7
7	F	27	IDU	1a	6.4–7.2	2	N/A	N/A	N/A	N/A	23	3	66	81	3.4
8	F	15	BT, IDU	1b	5.6–6.3	32	1	68	32	3.0	37	1	44	35	3.6
9	F	32	Others	1a	5.7–6.8	10	1	38	74	1.7	15	1	41	63	1.7

a IDU, intravenous drug use; BT, blood transfusion; others include tattooing, body piercing, sharing personal care items, blood-to-blood contact during sexual activity, intranasal cocaine use, and unknown.

b HCV RNA range (log_10_ IU/ml) was estimated as the range of all documented RNA titers of the archived specimens until the late time point for each participant.

c AST, ALT and AFP levels were estimated as the mean of all documented enzymatic levels of the year of the early or late time points for each participant.

### Cross-sectional genome-wide diversity increased during chronic infection

**Figure 2:**
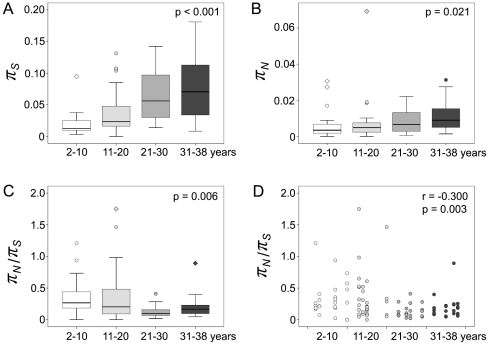
Nucleotide diversity compared according to the estimated year of HCV infection. The value of each amplified fragment covering HCV genome was considered as an independent data point. Panel A, synonymous nucleotide diversity (*π_S_*). The distribution of *π_S_* differed significantly across the estimated decades of infection (p<0.001). Panel B, non-synonymous nucleotide diversity (*π_N_*). The distribution of *π_N_* differed significantly across the estimated decades of infection (p = 0.021). Panel C, *π_N_*/*π_S_* ratio. The distribution of *π_N_*/*π_S_* ratio differed significantly across the estimated decades of infection (p = 0.006). Panel D, *π_N_*/*π_S_* ratio. The *π_N_*/*π_S_* ratio was significantly negatively correlated to the estimated year of HCV infection (r = −0.300, p = 0.003).

Median *π_S_* (0.0317) was significantly greater than median *π_N_* (0.0052) over the near genome-wide HCV genome (p<0.001). Thus, purifying selection predominated in the regions of the genome analyzed. When the data were plotted according to the estimated years after infection, significant differences of the distribution of *π_S_*, *π_N_* and *π_N_*/*π_S_* were found among the four categories of 2–10 years, 11–20 years, 21–30 years and 31–38 years after infection (p<0.001 for *π_S_*, p = 0.021 for *π_N_*, and p = 0.006 for *π_N_*/*π_S_* respectively, Figure 2). *π_S_* and *π_N_* both increased over the decades during chronic infection, with a significant correlation to the estimated year of infection (r = 0.556 and p<0.001 for *π_S_*, r = 0.268 and p = 0.008 for *π_N_*). The median *π_N_*/*π_S_* differed significantly among the four categories, with the highest value (0.266) occurring at 2–10 years after infection, an intermediate value (0.203) at 11–20 years after infection, and the lowest values at 21–30 and 31–38 years after infection (0.096 and 0.166 respectively) (Figure 2C). In individual comparisons, the median *π_N_*/*π_S_* at 2–10 years after infection differed significantly from that at 31–38 years after infection (p = 0.034) and from that at 21–38 years after infection (p = 0.002). The decreased *π_N_*/*π_S_* ratio was negatively correlated to the estimated year of infection with a correlation coefficient of −0.300 at statistical significance (p = 0.003; Figure 2D).

### Cross-sectional genome-wide diversity associated with disease status

**Figure 3:**
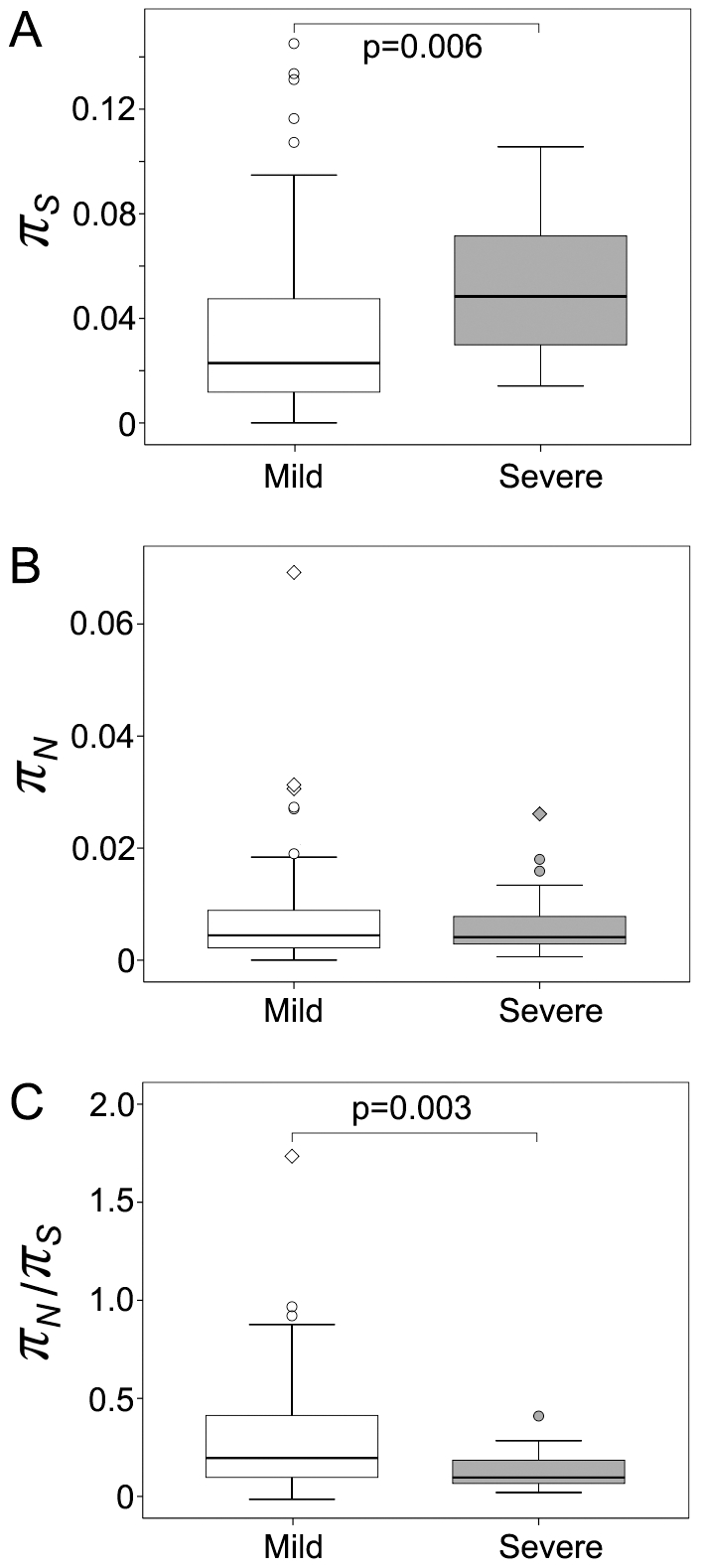
Nucleotide diversity compared according to disease status. The value of each amplified fragment covering HCV genome was considered as an independent data point. Panel A, synonymous nucleotide diversity (*π_S_*). The distribution of *π_S_* differed significantly between the mild and severe groups (p = 0.006). Panel B, non-synonymous nucleotide diversity (*π_N_*). No significant difference of *π_N_* was found between the mild and severe groups. Panel C, *π_N_*/*π_S_* ratio. The distribution of *π_N_*/*π_S_* differed significantly between the mild and severe groups (p = 0.003).

Fourteen out of the eighteen specimens were matched with a fibrosis stage (0–4) and a disease status (mild or severe) at the corresponding time point. Figure 3 shows the comparisons of *π_S_*, *π_N_* and *π_N_*/*π_S_* between the specimens from the mild or severe cases (n = 10 and n = 4 for mild and severe, respectively). The median *π_S_* in specimens from the mild cases was significantly lower than that from the severe cases (p = 0.006, Figure 3A), while the median *π_N_* did not differ significantly between the two groups (Figure 3B). The *π_N_*/*π_S_* ratio showed a wide range of variations in the group of mild cases, with a median value significantly greater than that from the severe cases (0.211 and 0.096 for mild and severe respectively, p = 0.003).

**Figure 4:**
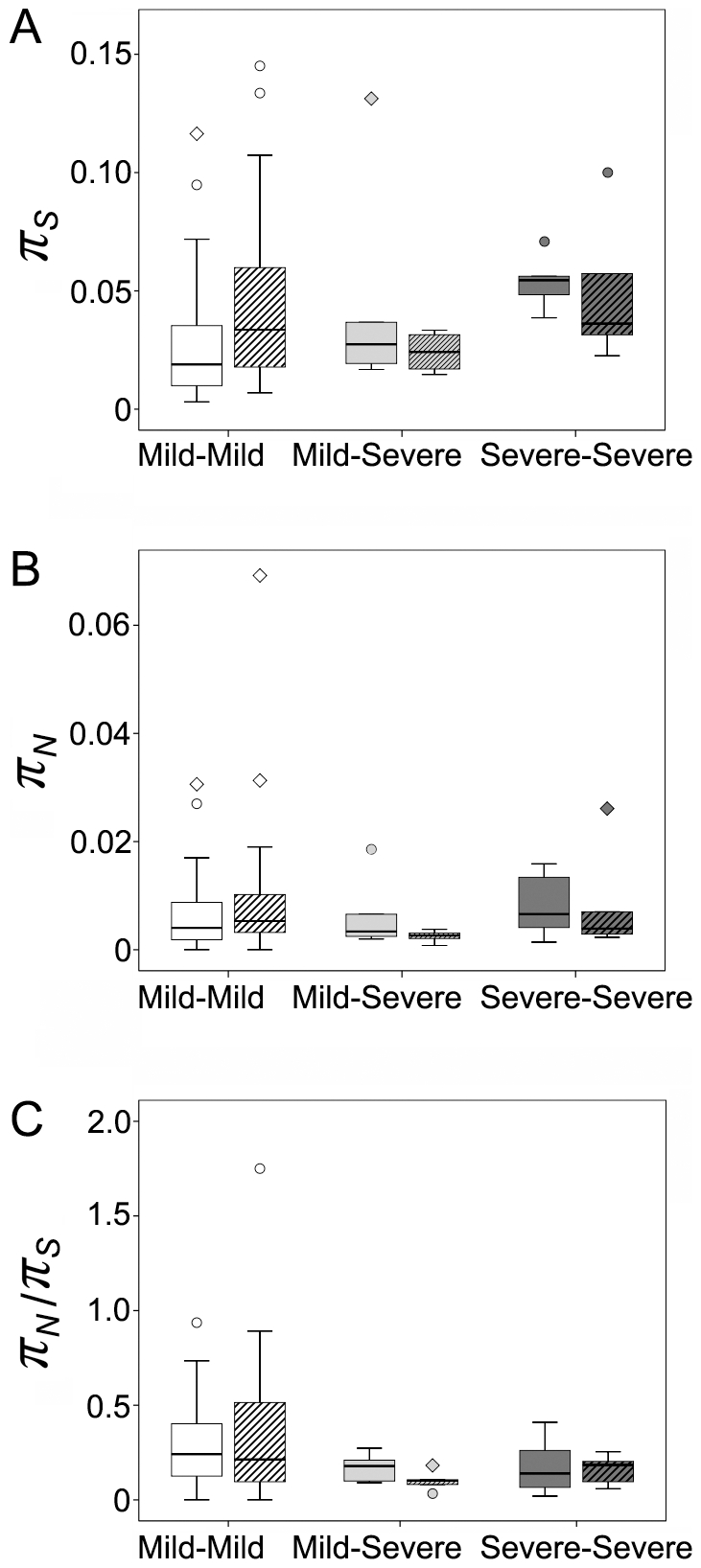
Nucleotide diversity compared between the early and late time points. The value of each amplified fragment covering HCV genome was considered as an independent data point. Panel A, synonymous nucleotide diversity (*π_S_*). Panel B, non-synonymous nucleotide diversity (*π_N_*). Panel C, *π_N_*/*π_S_* ratio. None of the comparisons showed significant difference between the early and late time points for the categories of participants with consistent mild, mild to severe, and consistent severe disease.

Six out of the nine patients had fibrosis stages available at both early and late serum collections. Figure 4 shows the comparisons of *π_S_*, *π_N_* and *π_N_*/*π_S_* between the early and late time points according to the changes of disease status (4 participants with consistent mild disease, 1 participant with conversion from mild to severe disease, 1 participant with consistent severe disease). *π_S_* and *π_N_* decreased in the participants with severe disease outcomes (mild-severe or severe-severe) and slightly increased in the participants with consistent mild disease, although the differences between early and late time points did not reach statistical significance (Figures 4A and 4B). The values of *π_N_*/*π_S_* were lower in the participants with severe outcomes, with the lowest mean value (0.096) at the late time point from the participant with mild to severe conversion (Figure 4C). The differences between the early and late time points were not statistically significant, which was not surprising given the limited sample size.

### Longitudinal genome-wide divergence associated with disease status

**Figure 5:**
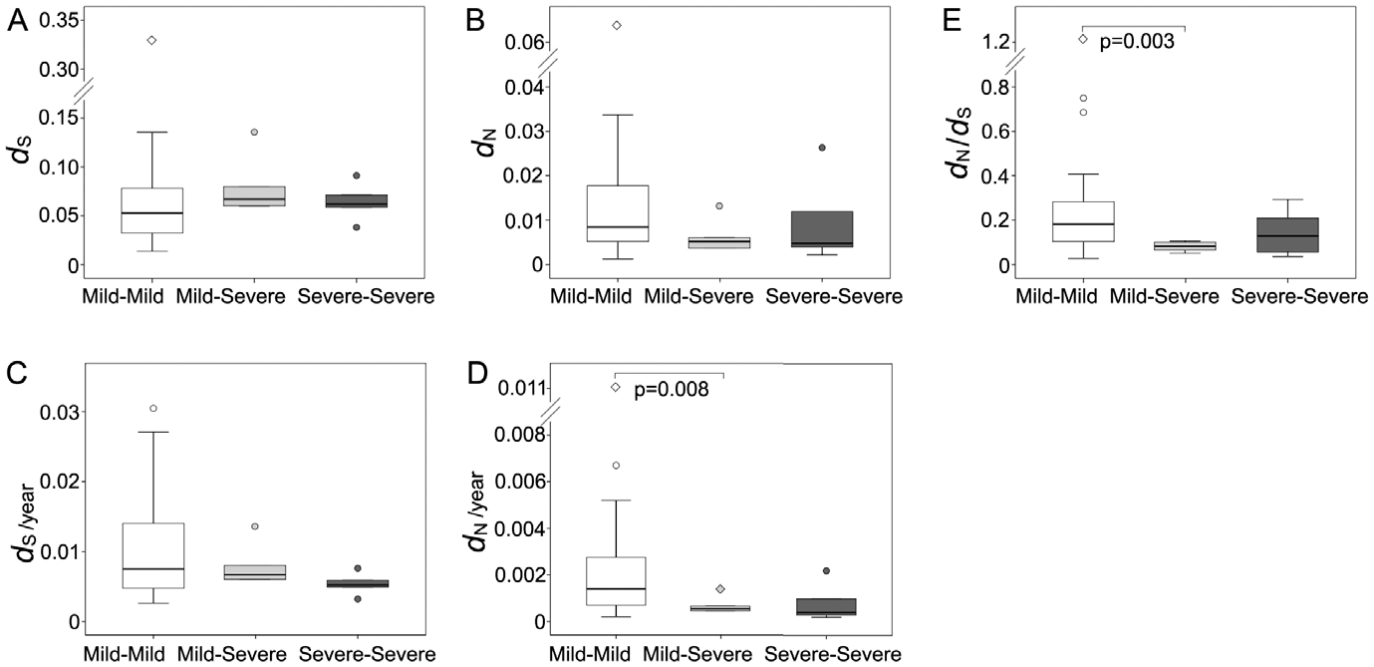
Nucleotide divergence compared according to disease status. The value of each amplified fragment covering HCV genome was considered as an independent data point. Panel A, synonymous nucleotide divergence (*d_S_*). Panel B, non-synonymous nucleotide divergence (*d_N_*). Panel C, synonymous nucleotide divergence per year (*d_S_*/year). Panel D, non-synonymous nucleotide divergence per year (*d_N_*/year). A significant difference of *d_N_*/year was found between the participants with consistent mild disease and those with mild to severe disease (p = 0.008). Panel E, *d_N_*/*d_S_* ratio. A significant difference of *d_N_*/*d_S_* ratio was found between the participants with consistent mild disease and those with mild to severe disease (p = 0.003).

Genome-wide divergence (*d_S_*, *d_N_*, and *d_N_*/*d_S_* ratio) was calculated between the sequences at early and late time points for the six participants with fibrosis stages available at both serum collections (4 participants with consistent mild disease, 1 participant with conversion from mild to severe disease, 1 participant with consistent severe disease). For all three categories, the median *d_S_* between early and late specimens was significantly greater than the median *d_N_* (p<0.001 for the mild-mild cases, p = 0.002 for the mild-severe case, and p = 0.008 for the severe-severe case respectively, Figures 5A and 5B). Thus there was no evidence of positive selection favoring amino acid changes. Three participants (2, 4 and 7) were known as HLA-A2 positive, and nucleotide substitutions were tested for the specific CD8+ T cell epitopes presented by HLA-A2 molecules. The median *d_S_* was significantly greater than the median *d_N_* in the epitope region (data not shown), providing no evidence of positive selection favoring amino acid changes in the specific CD8+ T cell epitopes.

Overall, the distribution of nucleotide divergence (*d_S_* and *d_N_*) between the early and late time points did not differ significantly among the categories of disease status (Figures 5A and 5B). In comparison of the rate of nucleotide divergence (*d_S_*/year and *d_N_*/year, Figures 5C and 5D), the median of *d_N_*/year was significantly greater in the mild-mild cases than that in the mild-severe case (p = 0.008) or that in the two cases with severe outcomes (p = 0.002). The highest median of *d_N_*/*d_S_* ratio was found in the mild-mild cases (0.182), which was significantly greater than that in the mild-severe case (0.080, p = 0.003) and that in the two cases with severe outcomes (0.090, p = 0.011) (Figure 5E).

### Genome-wide and gene-specific amino acid variation

The Shannon entropy per site was determined from the deduced amino acid sequences of the open reading frame of each amplified fragment as an indicator of the genome-wide amino acid variation. The intra-time-point Shannon entropy of amplified fragments differed at borderline significance among the estimated decades of infection (p = 0.051) and increased in significant association with the estimated year of infection (r = 0.224 and p = 0.025) (data not shown). When plotted according to disease status, no significant difference of the intra-time-point Shannon entropy was found between the mild and severe groups (data not shown). Similarly, no significant difference of the inter-time-point Shannon entropy was found between the cases with consistent mild disease and those with severe outcomes (data not shown).

**Figure 6:**
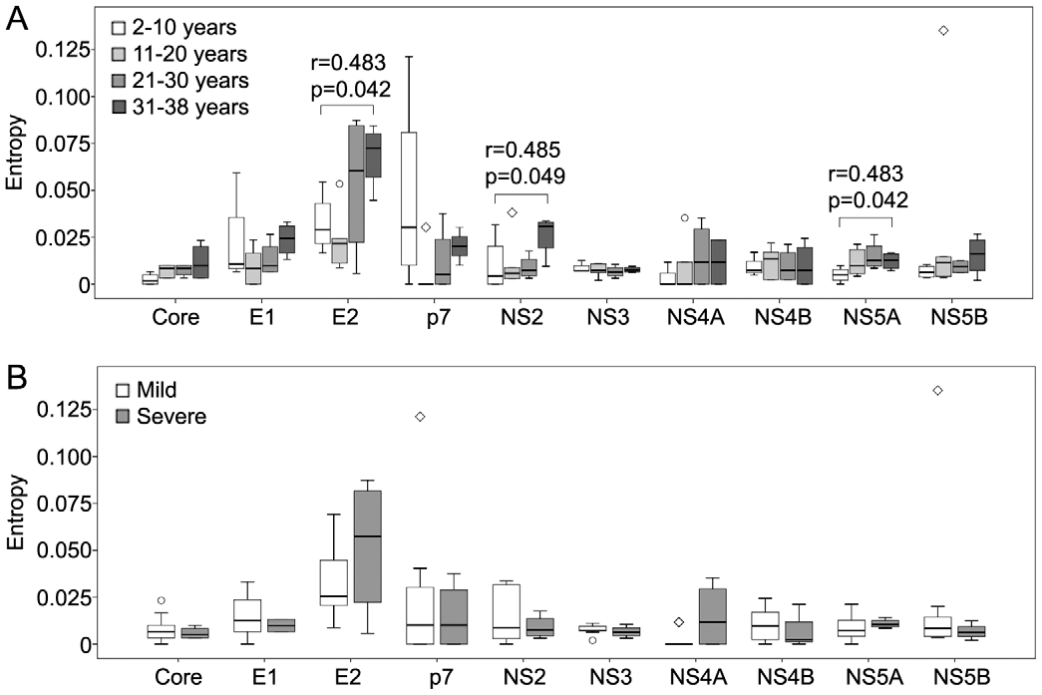
Intra-time-point Shannon entropy of individual genes. The Shannon entropy was calculated at single time point based on the amino acid sequences of individual genes. Panel A, the intra-time-point Shannon entropy of each gene compared according to the estimated year of infection. A significant correlation between the Shannon entropy and the estimated year of infection was found for E2, NS2, and NS5A genes. Panel B, the intra-time-point Shannon entropy of each gene compared according to the mild and severe disease status. No significant difference was found for the genes between the two disease groups.

To analyze gene-specific amino acid variation, the Shannon entropy per site was determined for each individual gene. The envelope gene E2 displayed the highest values of the intra-time-point Shannon entropy, which were significant different from the values of the other genes (p<0.001). When plotted according to duration of infection, the values of intra-time-point Shannon entropy of three genes (E2, NS2 and NS5A) showed significant correlation to the estimated year of infection (r = 0.483 and p = 0.042 for E2, r = 0.485 and p = 0.049 for NS2, and r = 0.483 and p = 0.042 for NS5A, respectively, Figure 6A). When plotted according to disease status, no difference of the intra-time-point Shannon entropy was found between the mild and severe cases for the individual genes (Figure 6B).

**Figure 7:**
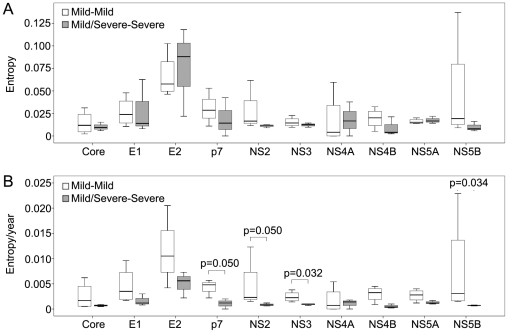
Inter-time-point Shannon entropy of individual genes. The Shannon entropy was calculated between the early and late time points based on the amino acid sequences of individual genes. Panel A, the inter-time-point Shannon entropy of each gene compared between the participants with mild or severe outcomes. No significant difference was found for the genes between the two disease groups. Panel B, yearly change of the inter-time-point Shannon entropy compared between the participants with mild or severe outcomes. Significant differences were found for the genes of p7, NS2, NS3, and NS5B between the two disease groups.

The values of inter-time-point Shannon entropy were plotted in Figure 7 according to the disease outcomes (consistent mild disease vs. severe disease). Although the inter-time-point Shannon entropy did not differ significantly between the two groups, the yearly change of the inter-time-point Shannon entropy of four nonstructural genes were significantly higher in the cases with consistent mild disease than those with severe outcomes (p = 0.050 for p7, p = 0.050 for NS2, p = 0.032 for NS3, and p = 0.034 for NS5B, respectively, Figure 7B).

## Discussion

Persistence of viremia with diversification of viral genomes is a hallmark of chronic HCV infection [9], while insidious course with variable presentation and progression is a hallmark of the associated hepatitis C disease [3]. Immunity appears to play a major role in control of HCV in the acute phase, as subjects who resolve acute HCV infection have more robust and broader specificity T cell responses compared to subjects unable to resolve acute infection [28]. In the chimpanzee model, elegant immune depletion studies have confirmed the role of T cells immunity in control of acute hepatitis C [29]. Viral mutational escape from immune pressure is known to occur, and is considered a major mechanism of HCV persistence [30], [31]. More recent research has also implicated two additional mechanisms of HCV persistence: escape from innate immunity via inhibitory action of viral products on host signaling [32], and exhaustion of virus-specific adaptive immune responses [33], [34], [35], which occurs during the early, post-acute phase of infection, and extends into the chronic phase of infection, and in theory, may reduce selection.

The role immune escape plays in HCV persistence during the chronic phase of infection is less clear, as are effects of immune exhaustion on HCV chronic persistence. The role of immunity in pressuring HCV during chronic hepatitis C, and in potentially causing HCV-associated liver injury, is an extremely important question, which greatly influences therapeutic vaccine development. Historically, the immunopathogenesis theory [36], which believes HCV-specific immunity causes the vast majority of liver injury, has dominated thinking on hepatitis C disease mechanisms. One way to estimate host pressure on HCV during the chronic phase of infection is via sequencing of viral genomes over time, and analyzing mutation patterns. The synonymous mutation frequency (*d_S_*) generally reflects viral replication efficiency, while non-synonymous mutations (*d_N_*) generally reflect effects of selection. *d_N_*/*d_S_* ratios greater than 1 reflect positive selection against virus (i.e., under immune pressure), while *d_N_*/*d_S_* ratios lower than 1 provide an index of purifying selection: the lower the *d_N_*/*d_S_* ratio, the stronger the purifying selection.

Toward the objective of characterizing HCV evolution during the natural disease course in humans, the present study derived near genome-wide sequences of HCV genomes that circulated over time in 9 chronically infected research subjects, all of whom were monoinfected with HCV genotype 1. This is the first long-term, population-based study of genome-wide viral diversity during chronic HCV infection, with all participants recruited from a well-characterized Alaska Native cohort, with specimens and clinical information archived for as long as 3 decades [9], [15]. The treatment naïve history of the 9 participants allowed investigation of the association between HCV diversity and disease development during the natural course of chronic infection, and to make inferences regarding positive versus neutral selection during virus evolution.

Examination of genome-wide nucleotide diversity showed an excess of synonymous substitutions per synonymous site over non-synonymous substitutions per non-synonymous site in the coding regions, a pattern seen in the analysis of both intra-time-point comparison and divergence between the early and late time points. The predominance of synonymous substitutions suggested that these sequences were subjected to strong purifying selection, acting to eliminate non-synonymous mutations. This pattern is opposite of what one would expect if immune pressure were a prime influence on evolution, in which case non-synonymous mutations would be favored. Rather, this observation supports the hypothesis of the neutral theory of molecular evolution, where purifying selection is ubiquitous in eliminating deleterious mutations, whether they be non-synonymous mutations resulting in amino acid changes, or synonymous mutations altering critical RNA secondary structure, for example [37]. The same pattern of purifying selection was seen even in HLA-A2 epitopes. Thus, although escape from CD8+ T cell recognition is recognized as a persistence mechanism during acute infection [30], the earliest samples analyzed here were generally taken several years after infection, when escape mutations likely have occurred and become fixed in the viral population infecting a given host [30].

One model to explain the cumulative data on HCV genetics and immunity is that, for chronic HCV infection to occur, virus must first survive pressure during the acute phase, either by mutation, or inhibition of critical intracellular signaling, or T cell exhaustion, or some combination. Later, during the chronic phase, profound immune dysfunction could result in purifying selection of HCV, with predominant synonymous mutations. However, non-synonymous mutations not eliminated by purifying selection might result in pathogenic viral variants that accelerate the progression of disease. An example of how this might work is provided by the studies of Pavio and colleagues [38], where HCV core variants isolated from hepatocellular carcinoma cells differed, in a highly significant fashion, in ability to modulate the TGFbeta response pathway, which is critical for maintenance of cell differentiation, compared to HCV core isolated from adjacent, non-cancerous liver. The molecular mechanism involved inhibition of the DNA-binding activity of the Smad3/4 transcription factor complex, through a direct interaction between the central domain (amino acids 59–126) of tumor-derived core and the MH1 DNA-binding domain of Smad3, thus preventing its binding to DNA, and presumably promoting cell transformation by providing, to clonally expanding cells, resistance to TGF-beta antiproliferative effects. More experiments addressing frequency and functional significance of natural variation in HCV proteins are clearly needed.

In the present study, purifying selection persisted during chronic infection, as indicated by the significant negative correlation between the *d_N_*/*d_S_* ratio and the estimated year of infection. When comparing HCV genetics between persons with mild and severe disease, the efficiency of purifying selection was significantly greater in severe compared to mild disease cases, with a lower median *d_N_*/*d_S_* ratio, and a higher median *d_S_*. The participants with severe disease also showed significantly lower *d_N_*/*d_S_* ratios when comparing the early and late time points, compared to participants with consistent mild disease. Despite the obvious shortcomings of small sample size, the present genetic study found fewer imprints of adoptive, cellular immune pressure (non-synonymous mutations) in persons with severe compared to mild disease, and one could not conclude, from the data, that cellular immunity is the driving force of hepatitis C disease, as the leading model presumes. In fact, it has recently been determined, in both cell culture [39], and a small animal model [40], that HCV can cause injury and death to hepatocytes, in the absence of cellular immunity. The significant increases in *d_S_* with severe disease, may have been allowed by immune exhaustion, and might be due to enhanced viral generation, but might also be explained by increased virus stability due to RNA secondary structure advantage, for example. Further studies are needed to test these possibilities.

The present data hint that immune pressure may have been greatest in mild disease (this is presently under study), and thus might be one reason for lower efficiency of purifying selection in mild disease cases. The viral species are expected to accumulate more deleterious non-synonymous mutations and as a result, to show greater amino acid variation. One measure of such amino acid variation is the Shannon entropy, which is a quantitative measure of sequence dissimilarity that incorporates both the frequencies and the number of variation [8], [41]. In this study, the genome-wide Shannon entropy is significantly correlated to the estimated year of infection and increased gradually over decades, suggesting enhanced amino acid polymorphism due to accumulated non-synonymous mutations. Analysis of individual genes further indicated that amino acid variation was not uniformly distributed over the genome-wide open reading frame. The E2 gene was significantly distinguished from the other genes with the highest amino acid variation in both intra-time-point and inter-time-point comparisons. Besides, the genes of p7, NS2, NS3 and NS5B showed significantly higher yearly change of the Shannon entropy between the early and late time points in the participants with mild disease outcomes than in those with severe disease outcomes, suggesting that these four non-structural genes experienced less efficient purifying selection in the mild disease cases than in the severe disease cases. The elevated fixation of slightly deleterious mutations in the non-structural genes might incur significant disadvantages to the replication of HCV variants, since these genes have all been shown to play essential roles in the viral life cycle [42]. Although the outcome of chronic hepatitis C is heavily influenced by host factors such as age, gender, alcohol abuse, obesity [43], and possibly host genetics, the enhanced polymorphism of viral genomes could partially account for the relatively weakened pathogenesis and slower disease progression in the participants with mild disease.

**Figure 8:**
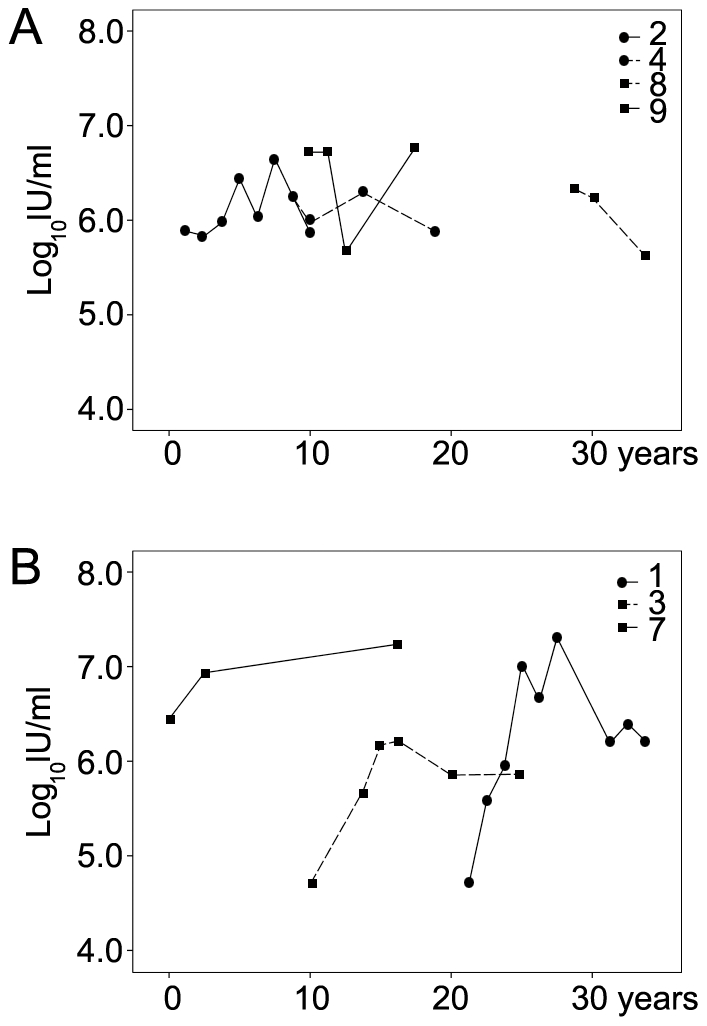
Fluctuation of serum HCV RNA level during chronic infection. Archived RNA levels (Log_10_ IU/mL) were plotted according to the estimated year of infection for individual participants. Panel A, HCV RNA levels in the participants with consistent mild disease. Panel B, HCV RNA levels in the participants with severe disease outcome.

According to the neutral theory, natural selection is more efficient in large populations. Mutations are affected mostly by random drift when the selection coefficient (s) is less than the reciprocal of the effective population size (|s|<1/N_e_) [44]. In a population with small effective size, the variants with small selection coefficients, particularly slightly deleterious mutations, will act as strictly neutral alleles to drift to higher frequencies and even to reach fixation [45], [46]. In the current study, the viral population sizes, as shown with the HCV RNA level in serum, fluctuated over time in the participants with either mild or severe disease outcomes (Figure 8). The participants with severe outcomes showed greater variation of viral loads compared to the mild cases (log_10_ IU/ml = 4.7–7.3 for the severe and 5.6–6.8 for the mild, respectively), and it is notable that the serum collections from the participants with severe outcomes were performed all at the peak platform of viral loads, and in the cases of 1 and 3, years after the viral breakthrough. Overall, the HCV viral loads were slightly higher in the severe cases compared to the mild cases (median = 6.20 vs. 6.13 respectively) and slightly increased according to the duration of infection (median = 6.03 for the first decade and 6.23 for the following decades). Interestingly, a previous study at a larger population scale from the same cohort of Alaska Native persons showed significant increasing viral loads according to the decades of infection [9]. The study also demonstrated that participants with mild disease had significantly greater changes in HCV RNA levels between specimens than did those with severe disease [9]. Since effective population size is nearer to the lower values than the higher values in the cases of fluctuating population sizes [37], the viral load data are consistent with the genetic evidence of more efficient purifying selection in severe disease.

Recently, bioinformatics and biophysical approaches have been applied to identify the configurations of viral genomic RNA, including the genome of HCV, and other mammalian RNA viruses. HCV showed evidence of genome-scale ordered RNA structure (GORS), identified as extensive internal base-pairing throughout the coding sequences, using a bioinformatics approach [47]. *In vitro* experiments further confirmed that HCV RNA transcripts were largely inaccessible to complimentary probes in hybridization solutions, and were maintained as tightly compacted structures during the deposition process under atomic force microscope [48]. Comparison among multiple viral families indicates that the presence of GORS is strongly associated with the ability of viral infections to persist in their natural hosts [47]. The maintenance of GORS is therefore an evolutionarily conserved feature, and hypothetically constrains the neutral drift of RNA virus sequences. It raises an intriguing possibility that the selection of synonymous and non-synonymous nucleotide substitutions within the HCV genome is not simply due to amino acid variation, but also due to nucleotide interactions in the secondary RNA structure [49], which we did not analyze for the present study.

In summary, the present study characterized the efficiency of purifying selection of HCV genomes using a variety of genetic traits including the nucleotide substitutions, the *d_N_*/*d_S_* ratio and the Shannon entropy of amino acid variations during chronic infection. Our results strongly indicate that the HCV genome has a significantly higher rate of accumulating synonymous substitutions relative to non-synonymous substitutions. The effective population size of the virus infecting an individual host appears to vary more substantially over time in the participants with mild disease, which results in an elevated fixation probability of slightly deleterious mutations due to less efficient purifying selection in small populations. The accumulation of deleterious mutations might incur a threat to pathogenesis and viral persistence. However, there is no correlation between the viral loads and the progression of liver disease, and direct evidence has not been available to assess the effect of slightly deleterious mutations on the biochemical function of the viral genes. Additional analysis is further needed to evaluate in detail the role of purifying selection and viral pathogenesis during chronic HCV infection. Likewise, the breadth of potential interactions between host and pathogen in this disorder needs further definition.

## Supporting Information

Figure S1 **Phylogenetic trees based on the Core sequences from the 9 participants (P1–9).** Clones 1–3 were obtained from the serum sample at the early time point of the corresponding participant, and clones 4–6 were obtained from the sample at the late time point. Panel A, phylogenetic tree based on the nucleotide sequences of Core. Panel B, Phylogenetic trees based on the predicted amino acid sequences of Core. The trees were constructed with the neighbor-joining method using the MacVector 9.5.1 software (MacVector Inc, Cary, NC). Reference sequences with Accession Numbers (1a, 1b, 2a and 2b) are included as outgroups and to identify the corresponding genotype. The scale bar indicates that the horizontal branch length represents 2 nucleotide (Panel A) or 1 (Panel B) amino acid substitutions per 100 sites.
(TIF)

Table S1 **PCR primers and amplification strategy.**
(DOC)

Table S2 **Sequencing primers of HCV genome.**
(DOC)

Text S1 **The review boards that approved this study and the corresponding dates.**
(DOC)
