# Supplementary figures and images for: Genetic Diversity of Near Genome-Wide Hepatitis C Virus Sequences during Chronic Infection: Evidence for Protein Structural Conservation Over Time
Source: PLoS One. 2011 May 5;6(5):e19562. doi: 10.1371/journal.pone.0019562 (PMC3088699; doi:10.1371/journal.pone.0019562)

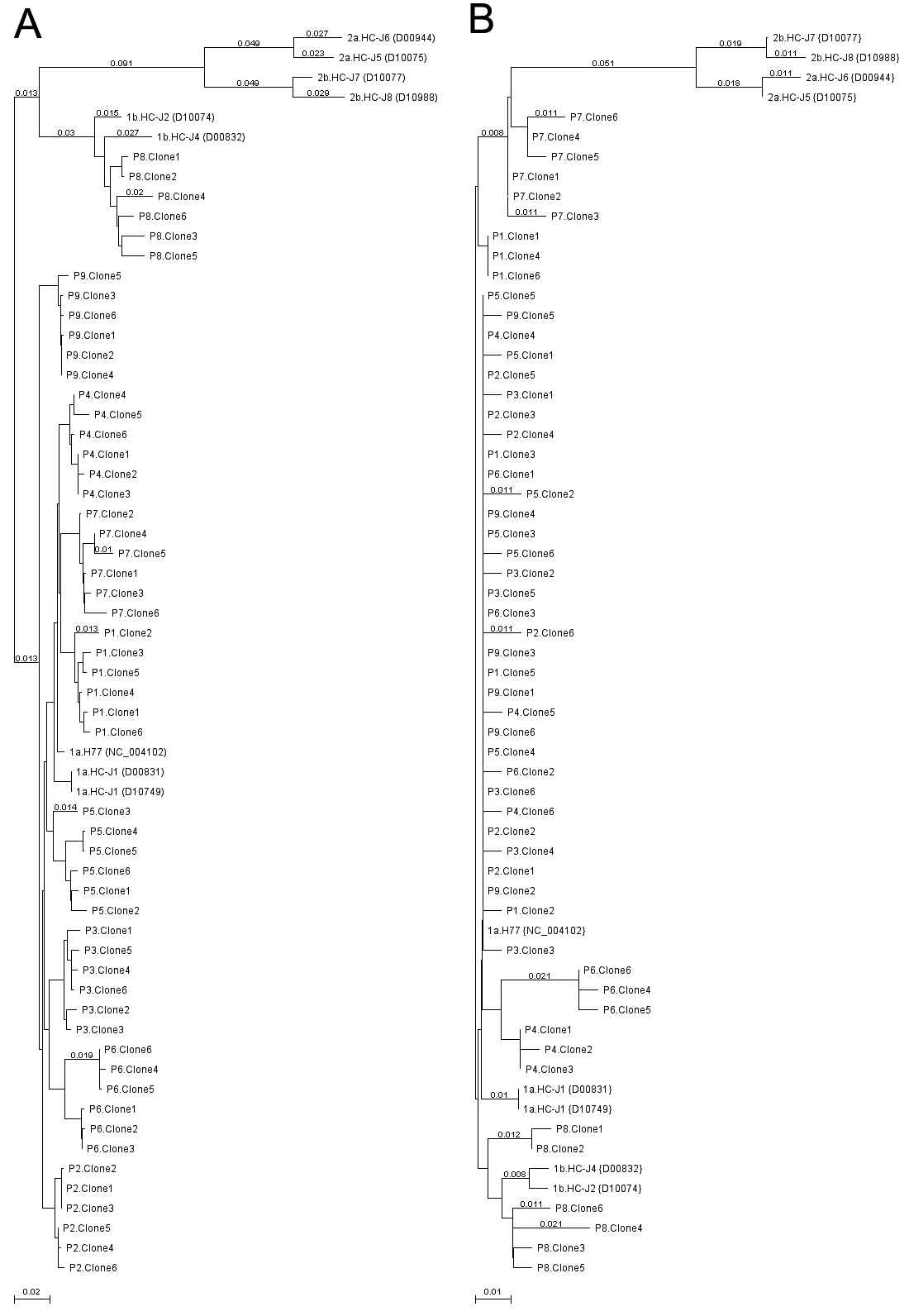

Supplement: Figure S1 — Phylogenetic trees based on the Core sequences from the 9 participants (P1–9). Clones 1–3 were obtained from the serum sample at the early time point of the corresponding participant, and clones 4–6 were obtained from the sample at the late time point. Panel A, phylogenetic tree based on the nucleotide sequences of Core. Panel B, Phylogenetic trees based on the predicted amino acid sequences of Core. The trees were constructed with the neighbor-joining method using the MacVector 9.5.1 software (MacVector Inc, Cary, NC). Reference sequences with Accession Numbers (1a, 1b, 2a and 2b) are included as outgroups and to identify the corresponding genotype. The scale bar indicates that the horizontal branch length represents 2 nucleotide (Panel A) or 1 (Panel B) amino acid substitutions per 100 sites. (TIF) [file pone.0019562.s001.tif]
