# Supplementary material for: Genetic Diversity of Near Genome-Wide Hepatitis C Virus Sequences during Chronic Infection: Evidence for Protein Structural Conservation Over Time
Source: PLoS One. 2011 May 5;6(5):e19562. doi: 10.1371/journal.pone.0019562 (PMC3088699; doi:10.1371/journal.pone.0019562)
Supplement: Table S1 — PCR primers and amplification strategy. (DOC) [file pone.0019562.s002.doc]

**Table S1. PCR primers and amplification strategy.**

| Patient ID | PCR Fragment | Primer | Sequence (5’-3’) | Binding Site | Accession number |
| --- | --- | --- | --- | --- | --- |
| 1 | A | 0031S | CATGAATCACTCCCCTGTGAGG | 31-52 | HQ113464-69 |
|  |  | 1600AS | AGGCTTTCATTGCAGTTCAAGGCCGTGCTATTGATGTGCC | 1639-1600 |  |
| 1 | B | 1300S | GGCATGGGATATGATGATGAACTGGTCCCCTAC | 1295-1327 | HQ113470-75 |
|  |  | 3060AS | GCAAACTGGCTTGAAGAATC | 3060-3041 |  |
| 1 | C | 3027S | TTCGGACCCCTTTGGATTC | 3027-3045 | HQ113476-81 |
|  |  | 5027AS | AAAGACGCCCTCCCAAAATTC | 5027-5007 |  |
| 1 | D | 4677S | GGCGACTTCGACTCTGTGATAGAC | 4677-4700 | HQ113482-87 |
|  |  | 6682AS | CACGGGCATTTAAGATTGTCAGTAG | 6682-6658 |  |
| 1 | E | 6509S | GAGTGGGACGTTCCCCATTAAC | 6509-6530 | HQ113488-93 |
|  |  | 8583AS | CCGCACTTTCACAGATAACGACTAAG | 8583-8558 |  |
| 2 | A | 0031S | CATGAATCACTCCCCTGTGAGG | 31-52 | HQ113494-99 |
|  |  | 2076AS | CAATCAGTGGGGCAGTGCAG | 2095-2076 |  |
| 2 | B | 2076S | TTGCTCTGCCCCACTGATTG | 2076-2095 | HQ113500-05 |
|  |  | 3510AS | CCTCCACTTGGTTTTTGTC | 3510-3492 |  |
| 2 | C | 2539S | CCTGCTTGTGGATGATGTTAC | 2539-2559 | HQ113506-11 |
|  |  | 5100AS | CCAGGTAAGGAAAGTTCTCC | 5100-5081 |  |
| 2 | D | 4677S | GGCGACTTCGACTCTGTGATAGAC | 4677-4700 | HQ113512-17 |
|  |  | 6682AS | CACGGGCATTTAAGATTGTCAGTAG | 6682-6658 |  |
| 2 | E | 6509S | GAGTGGGACGTTCCCCATTAAC | 6509-6530 | HQ113518-23 |
|  |  | 8583AS | CCGCACTTTCACAGATAACGACTAAG | 8583-8558 |  |
| 3 | A | 0031S | CATGAATCACTCCCCTGTGAGG | 31-52 | HQ113524-29 |
|  |  | 1600AS | AGGCTTTCATTGCAGTTCAAGGCCGTGCTATTGATGTGCC | 1639-1600 |  |
| 3 | B | 1428S | TCCATGGTGGGGAACTGGGC | 1428-1447 | HQ113530-35 |
|  |  | 2076AS | CAATCAGTGGGGCAGTGCAG | 2095-2076 |  |
| 3 | C | 2076S | TTGCTCTGCCCCACTGATTG | 2076-2095 | HQ113536-41 |
|  |  | 3510AS | CCTCCACTTGGTTTTTGTC | 3510-3492 |  |
| 3 | D | 2539S | CCTGCTTGTGGATGATGTTAC | 2539-2559 | HQ113542-47 |
|  |  | 5100AS | CCAGGTAAGGAAAGTTCTCC | 5100-5081 |  |
| 3 | E | 4677S | GGCGACTTCGACTCTGTGATAGAC | 4677-4700 | HQ113548-53 |
|  |  | 6682AS | CACGGGCATTTAAGATTGTCAGTAG | 6682-6658 |  |
| 3 | F | 6509S | GAGTGGGACGTTCCCCATTAAC | 6509-6530 | HQ113554-59 |
|  |  | 8583AS | CCGCACTTTCACAGATAACGACTAAG | 8583-8558 |  |
| 4 | A | 0031S | CATGAATCACTCCCCTGTGAGG | 31-52 | HQ113560-65 |
|  |  | 1227AS | TGGGAGAGAAGGTAAACAG | 1227-1209 |  |
| 4 | B | 1203S | GGTCAACTGTTTACCTTCTCTC | 1203-1224 | HQ113566-71 |
|  |  | 2191AS | TGCCAAAGCCTATACGGGTAGTC | 2191-2169 |  |
| 4 | C | 2076S | TTGCTCTGCCCCACTGATTG | 2076-2095 | HQ113572-76 |
|  |  | 3060AS | GCAAACTGGCTTGAAGAATC | 3060-3041 |  |
| 4 | D | 3027S | TTCGGACCCCTTTGGATTC | 3027-3045 | HQ113577-81 |
|  |  | 4481AS | GATAGCCTTGCCGTAAAAAG | 4481-4462 |  |
| 4 | E | 3912S | AAGGCGGTGGACTTTATCC | 3912-3930 | HQ113582-87 |
|  |  | 5100AS | CCAGGTAAGGAAAGTTCTCC | 5100-5081 |  |
| 4 | F | 4677S | GGCGACTTCGACTCTGTGATAGAC | 4677-4700 | HQ113588-93 |
|  |  | 6682AS | CACGGGCATTTAAGATTGTCAGTAG | 6682-6658 |  |
| 4 | G | 6509S | GAGTGGGACGTTCCCCATTAAC | 6509-6530 | HQ113594-99 |
|  |  | 7608AS | ACATTGAGCAGCACACGAC | 7608-7590 |  |
| 4 | H | 7592S | CGTGTGCTGCTCAATGTCTTATTC | 7592-7615 | HQ113600-05 |
|  |  | 8583AS | CCGCACTTTCACAGATAACGACTAAG | 8583-8558 |  |
| 5 | A | 0031S | CATGAATCACTCCCCTGTGAGG | 31-52 | HQ113606-11 |
|  |  | 1600AS | AGGCTTTCATTGCAGTTCAAGGCCGTGCTATTGATGTGCC | 1639-1600 |  |
| 5 | B | 1600S | GGCACATCAATAGCACGGCCTTGAACTGCAATGAAAGCCT | 1600-1639 | HQ113612-17 |
|  |  | 3060AS | GCAAACTGGCTTGAAGAATC | 3060-3041 |  |
| 5 | C | 2539S | CCTGCTTGTGGATGATGTTAC | 2539-2559 | HQ113618-23 |
|  |  | 4481AS | GATAGCCTTGCCGTAAAAAG | 4481-4462 |  |
| 5 | D | 4677S | GGCGACTTCGACTCTGTGATAGAC | 4677-4700 | HQ113624-29 |
|  |  | 6682AS | CACGGGCATTTAAGATTGTCAGTAG | 6682-6658 |  |
| 5 | E | 6509S | GAGTGGGACGTTCCCCATTAAC | 6509-6530 | HQ113630-35 |
|  |  | 8583AS | CCGCACTTTCACAGATAACGACTAAG | 8583-8558 |  |
| 6 | A | 0031S | CATGAATCACTCCCCTGTGAGG | 31-52 | HQ113636-41 |
|  |  | 2076AS | CAATCAGTGGGGCAGTGCAG | 2095-2076 |  |
| 6 | B | 2076S | TTGCTCTGCCCCACTGATTG | 2076-2095 | HQ113642-47 |
|  |  | 4481AS | GATAGCCTTGCCGTAAAAAG | 4481-4462 |  |
| 6 | C | 3912S | AAGGCGGTGGACTTTATCC | 3912-3930 | HQ113648-53 |
|  |  | 4677AS | GTCTATCACAGAGTCGAAGTCGCC | 4700-4677 |  |
| 6 | D | 4677S | GGCGACTTCGACTCTGTGATAGAC | 4677-4700 | HQ113654-59 |
|  |  | 6682AS | CACGGGCATTTAAGATTGTCAGTAG | 6682-6658 |  |
| 6 | E | 6509S | GAGTGGGACGTTCCCCATTAAC | 6509-6530 | HQ113660-65 |
|  |  | 8583AS | CCGCACTTTCACAGATAACGACTAAG | 8583-8558 |  |
| 7 | A | 0031S | CATGAATCACTCCCCTGTGAGG | 31-52 | HQ113666-71 |
|  |  | 2076AS | CAATCAGTGGGGCAGTGCAG | 2095-2076 |  |
| 7 | B | 2076S | TTGCTCTGCCCCACTGATTG | 2076-2095 | HQ113672-77 |
|  |  | 3510AS | CCTCCACTTGGTTTTTGTC | 3510-3492 |  |
| 7 | C | 3497S | AAACCAAGTGGAGGGTGAGG | 3497-3516 | HQ113678-83 |
|  |  | 5100AS | CCAGGTAAGGAAAGTTCTCC | 5100-5081 |  |
| 7 | D | 4677S | GGCGACTTCGACTCTGTGATAGAC | 4677-4700 | HQ113684-89 |
|  |  | 6682AS | CACGGGCATTTAAGATTGTCAGTAG | 6682-6658 |  |
| 7 | E | 6509S | GAGTGGGACGTTCCCCATTAAC | 6509-6530 | HQ113690-95 |
|  |  | 8583AS | CCGCACTTTCACAGATAACGACTAAG | 8583-8558 |  |
| 8 | A | 0031S | CATGAATCACTCCCCTGTGAGG | 31-52 | HQ113696-701 |
|  |  | 1600AS | AGGCTTTCATTGCAGTTCAAGGCCGTGCTATTGATGTGCC | 1639-1600 |  |
| 8 | B | 1600S | GGCACATCAATAGCACGGCCTTGAACTGCAATGAAAGCCT | 1600-1639 | HQ113702-07 |
|  |  | 3337AS | CCGTTGATGATGTCACCGCA | 3337-3318 |  |
| 8 | C | 2535S | TGCTCCTGCTTGTGGATGATG | 2535-2555 | HQ113708-13 |
|  |  | 4677AS | GTCTATCACAGAGTCGAAGTCGCC | 4700-4677 |  |
| 8 | D | 4677S | GGCGACTTCGACTCTGTGATAGAC | 4677-4700 | HQ113714-19 |
|  |  | 6682AS | CACGGGCATTTAAGATTGTCAGTAG | 6682-6658 |  |
| 8 | E | 6509S | GAGTGGGACGTTCCCCATTAAC | 6509-6530 | HQ113720-25 |
|  |  | 8583AS | CCGCACTTTCACAGATAACGACTAAG | 8583-8558 |  |
| 9 | A | 0031S | CATGAATCACTCCCCTGTGAGG | 31-52 | HQ113726-31 |
|  |  | 1300AS | GACCAGTTCATCATCATATCCCATGCC | 1321-1295 |  |
| 9 | B | 0800S | GCGTCCGGGTTCTGGAAGACGGCGTGAACTATGCAACAGG | 802-841 | HQ113732-37 |
|  |  | 1600AS | AGGCTTTCATTGCAGTTCAAGGCCGTGCTATTGATGTGCC | 1639-1600 |  |
| 9 | C | 1300S | GGCATGGGATATGATGATGAACTGGTCCCCTAC | 1295-1327 | HQ113738-43 |
|  |  | 2670AS | ACCAYGCAAAGCAGAAGAAC | 2670-2651 |  |
| 9 | D | 2471S | GTCCTGGRCCATYAAGTGG | 2471-2489 | HQ113744-49 |
|  |  | 5039AS | RGTGAGGCCYGTAAAGACG | 5039-5021 |  |
| 9 | E | 4677S | GGCGACTTCGACTCTGTGATAGAC | 4677-4700 | HQ113750-55 |
|  |  | 6682AS | CACGGGCATTTAAGATTGTCAGTAG | 6682-6658 |  |
| 9 | F | 6183S | CTCAGCAGCCTCACTGTAAC | 6183-6202 | HQ113756-61 |
|  |  | 8188AS | GAGTATTGGAATCCGTAGGAG | 8188-8168 |  |

Note: The binding positions of primers are based on the sequence of HCV H77 strain (Genbank assession no. NC_004102). Primers were designed using the MacVector program (version 9.5, MacVector Inc., Cary, NC). The first three accession numbers are of the sequences at the early time point, and the following three (two for participant 4, fragments C and D) accession numbers are of the sequences at the late time point.
