# Supplementary material for: Genetic Diversity of Near Genome-Wide Hepatitis C Virus Sequences during Chronic Infection: Evidence for Protein Structural Conservation Over Time
Source: PLoS One. 2011 May 5;6(5):e19562. doi: 10.1371/journal.pone.0019562 (PMC3088699; doi:10.1371/journal.pone.0019562)
Supplement: Table S2 — Sequencing primers of HCV genome. (DOC) [file pone.0019562.s003.doc]

**Table S2. Sequencing primers of HCV genome.**

| Primers | Orientation | Sequence (5’-3’) | Binding Site |
| --- | --- | --- | --- |
| 344S | Sense | 5'- GAGCACGAATCCTAAACC -3' | 344-361 |
| 456AS | Antisense | 5'- GCGGCAACAAGTAAACTC -3' | 456-439 |
| 817S | Sense | 5'- AAGACGGCGTGAACTATGC -3' | 817-835 |
| 866AS | Antisense | 5'- AGAGAAAGAGCAACCAGG -3' | 866-849 |
| 1203S | Sense | 5'- GGTCAACTGTTTACCTTCTCTC -3' | 1203-1224 |
| 1227AS | Antisense | 5'- TGGGAGAGAAGGTAAACAG -3' | 1227-1209 |
| 1720S | Sense | 5'- GACGCCTTACCGATTTTG -3' | 1720-1737 |
| 1737AS | Antisense | 5'- CAAAATCGGTAAGGCGTC -3' | 1737-1720 |
| 2515AS | Antisense | 5'- AGGAGGAACAGGAGAACGAC -3' | 2515-2496 |
| 3027S | Sense | 5'- TTCGGACCCCTTTGGATTC -3' | 3027-3045 |
| 3060AS | Antisense | 5'- GCAAACTGGCTTGAAGAATC -3' | 3060-3041 |
| 3497S | Sense | 5'- AAACCAAGTGGAGGGTGAGG -3' | 3497-3516 |
| 3510AS | Antisense | 5'- CCTCCACTTGGTTTTTGTC -3' | 3510-3492 |
| 3912S | Sense | 5'- AAGGCGGTGGACTTTATCC -3' | 3912-3930 |
| 3942AS | Antisense | 5'- GGTTCTCCACAGGGATAAAG -3' | 3942-3923 |
| 4481AS | Antisense | 5'- GATAGCCTTGCCGTAAAAAG -3' | 4481-4462 |
| 4528S | Sense | 5'- CAAAGAAGAAGTGCGACG -3' | 4528-4545 |
| 5151S | Sense | 5'- TGGGACCAGATGTGGAAGTG -3' | 5151-5170 |
| 5672AS | Antisense | 5'- GCCCGCCAAGTATTGTATC -3' | 5672-5654 |
| 5674S | Sense | 5'- TGTCAACGCTGCCTGGTAAC -3' | 5674-5693 |
| 6183S | Sense | 5'- CTCAGCAGCCTCACTGTAAC -3' | 6183-6202 |
| 6198AS | Antisense | 5'- CAGTGAGGCTGCTGAGTATG -3' | 6198-6179 |
| 7063S | Sense | 5'- TCACCAGGGTTGAGTCAGAG -3' | 7063-7082 |
| 7079AS | Antisense | 5'- TGACTCAACCCTGGTGATG -3' | 7079-7061 |
| 7592S | Sense | 5'- CGTGTGCTGCTCAATGTCTTATTC -3' | 7592-7615 |
| 7608AS | Antisense | 5'- ACATTGAGCAGCACACGAC -3' | 7608-7590 |
| 8182S | Sense | 5'- AATACTCACCAGGACAGCGG -3' | 8182-8201 |
| 8188AS | Antisense | 5'- GAGTATTGGAATCCGTAGGAG -3' | 8188-8168 |

Note: The binding positions of primers are based on the sequence of HCV H77 strain (Genbank assession no. NC_004102). Primers were designed using the MacVector program (version 9.5, MacVector Inc., Cary, NC).
