# Supplementary material for: Genetic Diversity of Near Genome-Wide Hepatitis C Virus Sequences during Chronic Infection: Evidence for Protein Structural Conservation Over Time
Source: PLoS One. 2011 May 5;6(5):e19562. doi: 10.1371/journal.pone.0019562 (PMC3088699; doi:10.1371/journal.pone.0019562)
Supplement: Text S1 — The review boards that approved this study and the corresponding dates. (DOC) [file pone.0019562.s004.doc]

**Text S1. The review boards that approved this study and the corresponding dates.**

Alaska Area Institutional Review Board (IHS IRB #2)

Study Title: Viral Host Interaction in Hepatitis C

IRB Reference #: 2005-12-027

Approval Date: January 18, 2011

Expiration Date: January 17, 2012

Alaska Area Institutional Review Board (HIS IRB #2)

Study Title: A Longitudinal Study of Chronic Hepatitis C Infections among Alaska Natives

IRB Reference #: 2004-12-018

Approval Date: January 18, 2011

Expiration Date: January 17, 2012

Centers for Disease Control and Prevention (CDC) Institutional Review Board

Study Title: A longitudinal study of Hepatitis C Infections among Alaska Natives

IRB Reference #: 3795.0

Approval Date: April 12, 2010

Expiration Date: April 24, 2011

University of Washington Institutional Review Board (Human Subjects Division)

Study Title: Hepatitis C Virus Replication and Liver Injury in the Human Host

IRB Reference #: 17729

Approval Date: January 16, 2011

Expiration Date: January 15, 2012
